# Regulation of feather follicle development and *Msx2* gene SNP degradation in Hungarian white goose

**DOI:** 10.1186/s12864-022-09060-z

**Published:** 2022-12-12

**Authors:** Yupu Song, Chang Liu, Yuxuan Zhou, Guangyu Lin, Chenguang Xu, Petunia Msuthwana, Sihui Wang, Jingyun Ma, Fangming Zhuang, Xianou Fu, Yudong Wang, Tuoya Liu, Qianyan Liu, Jingbo Wang, Yujian Sui, Yongfeng Sun

**Affiliations:** 1 College of Animal Science and Technology, Jilin Agricultural University, Changchun, 130118 China; 2 Changchun Animal Husbandry Service, Changchun, 130062 China; 3 Jilin Provincial Animal Husbandry Information Center, Changchun, 130000 China; 4 Key Laboratory for Animal Production, Product Quality and Safety of Ministry of Education, Changchun, 130118 China

**Keywords:** Hungarian white goose, Feather follicle, Goose down, SNP

## Abstract

**Background:**

Hungarian white goose has excellent down production performance and was introduced to China in 2010. The growth and development of feather follicles has an important impact on down production. Goose feather follicles can be divided into primary and secondary feather follicles, both of which originate in the embryonic stage. *Msx2* (Msh Homeobox 2) plays a regulatory role in tissues and organs such as eyes, teeth, bones and skin. However, its regulatory mechanism on goose feather follicles development remains unclear.

**Results:**

*Msx2* gene first increased, then decreased and increased at the end (E13, E18, E23, E28) during embryonic feather follicle development, and the expression level was the highest at E18. The pEGFP-N1-Msx2 overexpression vector and si-Msx2 siRNA vector were constructed to transfect goose embryo dermal fibroblasts. The results showed that the cell viability of ov-Msx2 group was significantly increased, and the gene expression levels of *FGF5* and *TGF-β1* genes were significantly down-regulated (*P* < 0.05), the expressions of *PCNA*, *Bcl2*, *CDK1*, *FOXN1* and *KGF* genes were significantly up-regulated (*P* < 0.05). After transfection of siRNA vector, the cell viability of the si-Msx2 group was significantly decreased (*P* < 0.01) compared with the si-NC group. *TGF-β1* expression was significantly up-regulated (*P* < 0.05), *FGF5* expression was extremely significantly up-regulated (*P* < 0.01), while *PCNA*, *Bcl2*, *CDK1*, *FOXN1* and *KGF* gene expression was significantly down-regulated (*P* < 0.05). High-throughput sequencing technology was used to mine the exon SNPs of *Msx2*. A total of 11 SNP loci were screened, four of the SNPs located in exon 1 were missense mutations. The feather follicle diameter of the GC genotype at the G78C site is significantly larger than that of the other two genotypes.

**Conclusions:**

*Msx2* maybe inhibit the apoptosis of goose dermal fibroblasts and promotes their proliferation. G78C can be used as a potential molecular marker for downy Variety.

**Supplementary Information:**

The online version contains supplementary material available at 10.1186/s12864-022-09060-z.

## Background

As a high-grade natural filler, goose down is not only light in texture, but also has high thermal insulation properties. Feather follicles are important basis for the development of goose down [1]. The positive feathers of goose are formed by the development of primary feather follicles, and the down feathers are formed by the development of secondary feather follicles, both of which start in the embryonic stage [2]. The Hungarian white goose originates from the *Anser anser*, introduced to China since 2010, which not only grows rapidly in the early stage, but also has high downy production.

The Msx2 (Msh Homeobox 2) gene belongs to the homeobox gene and is a highly conserved transcription factor in the Msx gene family [3]. Studies have shown that Msx2 is involved in the transcriptional regulation of lens development during lens development and is in the upstream of the regulatory network. The knockout of the Msx2 gene affects the apoptosis of lens cells and hinders the normal development of the lens [4]. Liu found that Msx2 knockout inhibited the expression of enamel matrix proteins and enamel mineralization [5]. Taguiar Overexpression of Msx2 gene in mouse bone marrow mesenchymal stem cells was found to increase the expression of cell surface markers and promote cell proliferation and osteogenesis [6]. The study by Jiang showed that the Msx2 gene was specifically expressed at the site of skin appendage formation, and it played a regulatory role in the growth and development of the skin and skin appendages; in transgenic mice, overexpression of the Msx2 gene resulted in Abnormal development of epidermis and hair follicles in mice [7]. Hughes showed that Msx2-deficient mice were unable to induce wound-induced hair follicle regeneration, and found that Msx2 gene expression enables epithelial cells to play a regulatory role in the development of new hair follicles [8]. However, its regulatory mechanism on goose hair follicle development remains unclear.

SNP (Single Nucleotide Polymorphism, SNP) is an important class of genetic markers, which has the characteristics of large number, dimorphism, stable inheritance, and easy automated detection [9]. SNP detection and typing technology has been widely used in the fields of livestock and poultry genetics research, breed selection, and seed resource protection, which has promoted the development of the livestock and poultry industry [10].

To explore the regulatory effect of *Msx2* gene on the development of goose feather follicles, in this study, four important time points (E13, E18, E23, E28) of feather follicle growth and development in the embryonic period of Hungarian white goose were selected to summarize the expression of *Msx2* gene. Isolation, identification, and culture of goose embryo dermal fibroblasts to extracted for functional verification of *Msx2* at the cellular level. Subsequently, using the *Msx2* sequence of Zhedong white goose (*Anser cygnoides*) genome database published in NCBI (NW_013185785.1) as a reference, the SNP of the Hungarian white goose *Msx2* exon was detected and typed. To preliminary explore the mechanism and molecular mechanism of *Msx2* in the regulation of goose feather follicle development and provide molecular markers and data support for the breeding of downy goose breeds.

## Results

### HE staining observation of feather follicles development in goose embryos

**Fig. 1:**
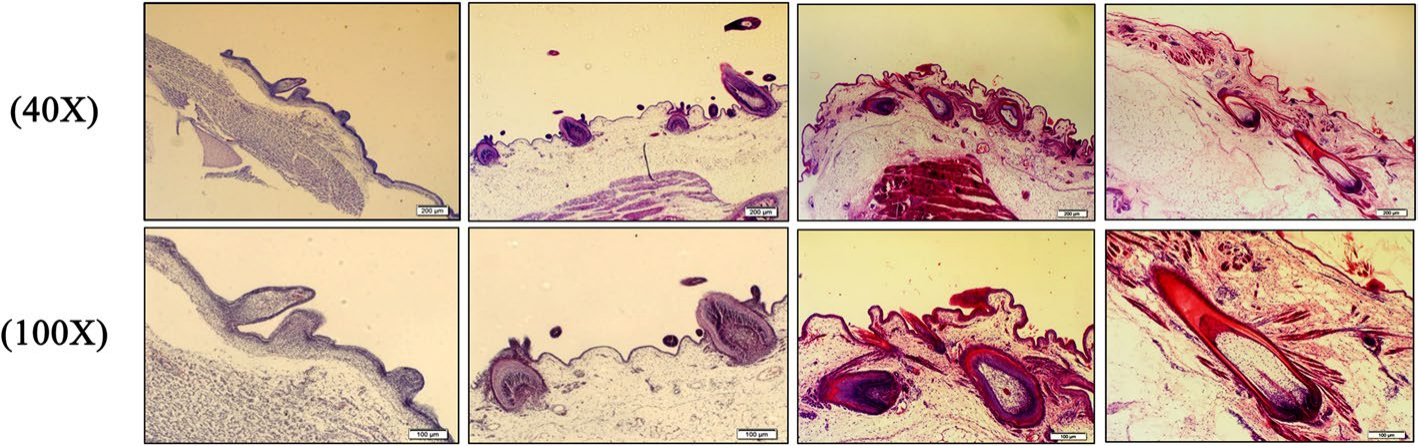
Observation on the morphology of feather follicles in goose Embryonic Stage. (The first line: Observation of feather follicle development in different embryonic stages under 40 times magnification, bar = 200 μm; The second row: Observation of feather follicle development in different embryonic stages under 100 times magnification, bar = 100 μm)

The histological sections of the morphogenesis of feather follicles in Hungarian white goose at four embryonic developmental stages (E13, E18, E23 and E28) were observed (Fig.1). The results showed that, on E13 (early embryonic development), the dorsal skin of the goose embryo forms a raised structure and feather buds appear, which is the stage of primary feather follicle formation. When the goose embryo develops to E18, With the continuous development and growth of the embryo, the dorsal skin of the goose embryo is keratinized, and the feather follicle structure can be seen in the visual field. The distance between the feather follicles is relatively close, and this is the secondary feather follicle developmental period. At E23, large primary feather follicles and secondary feather follicles can be observed simultaneously, which is the co-development period of primary and secondary feather follicles. At E28, the embryo development tends to mature, the degree of keratinization of the feathers is further deepened, and the typical feather follicle structure can be seen in the section. A few feather follicles can be seen, and various skin structures have gradually differentiated and matured.

### Relative expression of *Msx2* gene at important time points of feather follicle development in Hungarian white goose embryos

**Fig. 2:**
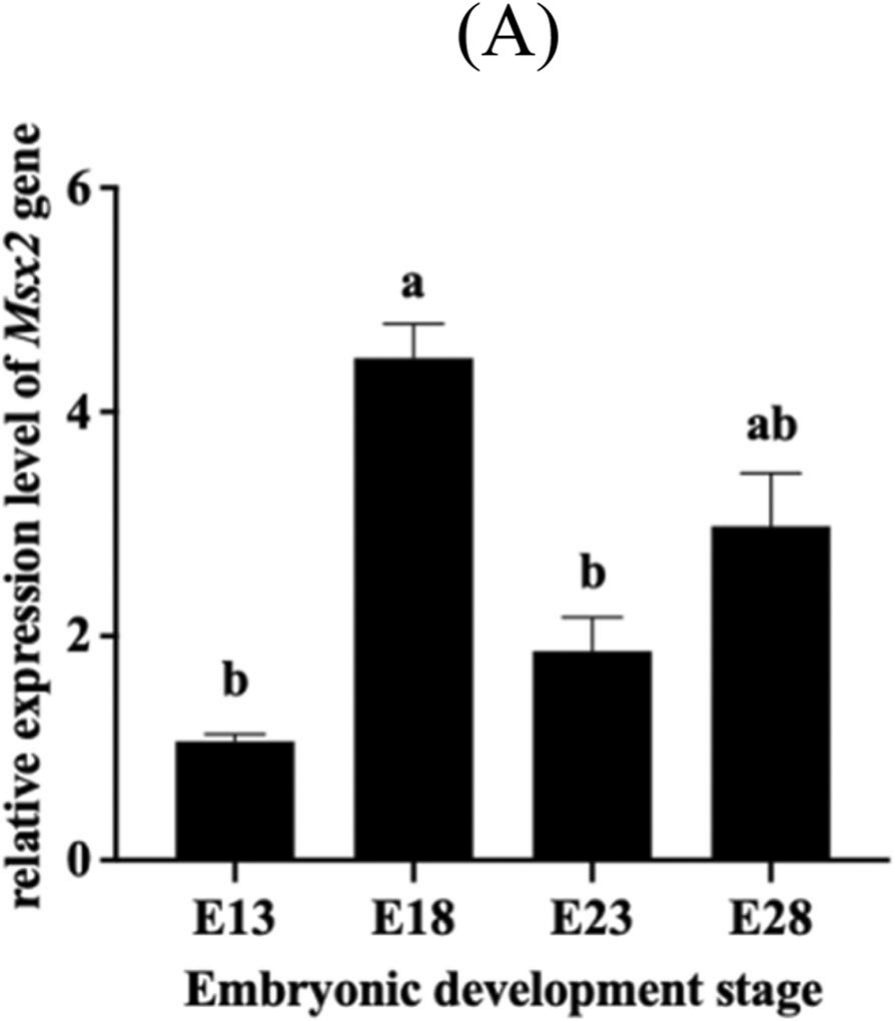
The relative expression of *Msx2* gene mRNA in the embryonic stage of Hungarian white goose. (Duncan multiple range test was used to determine statistical significance; Different letters indicate significant differences, *P* < 0.05, *n* = 3)

From the results of Quantitative Real-time PCR (Fig. 2), we can conclude that the relative expression of *Msx2* in the dorsal feather follicles of Hungarian white goose in the embryonic stage showed a trend of first increase, then decrease and then increase, among which the expression level was the lowest at E13 and the highest at E18. There was a significant difference in expression between E13 and E18 (*P* < 0.05), but no significant difference between E23 and E28 (*P* > 0.05).

### Identification of goose embryo dermal fibroblasts

The results of Giemsa staining and immunofluorescence detection confirmed that the cells isolated and cultured in this experiment were dermal fibroblasts.

### Construction of *Msx2* gene overexpression vector

**Fig. 3:**
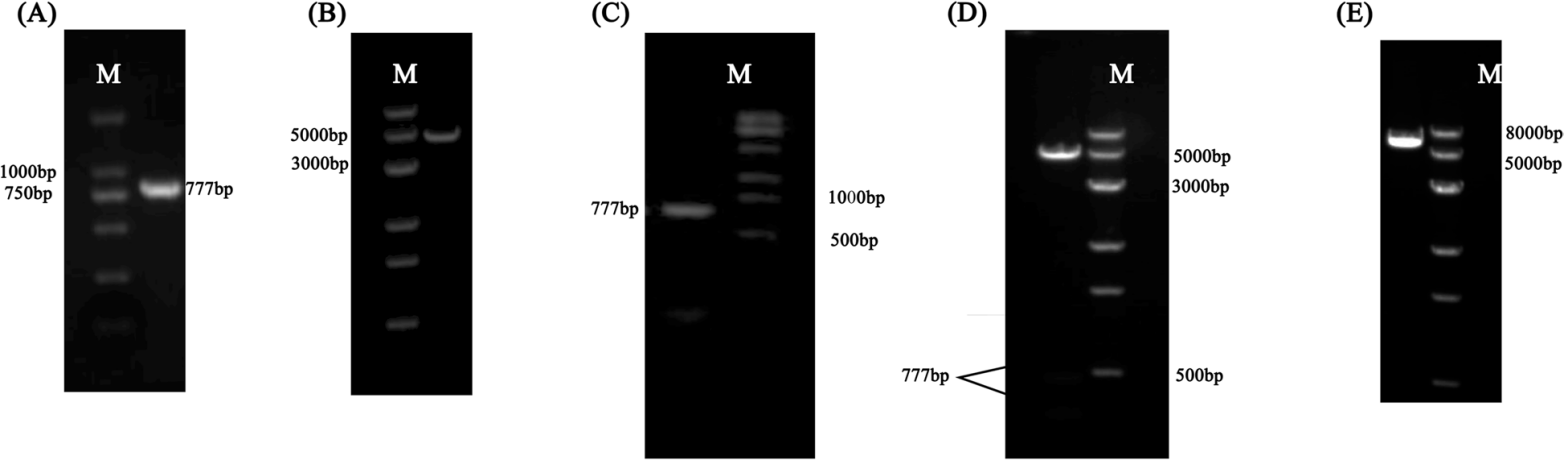
Construction of overexpression vector. (**A**: *Msx2* gene amplification, Marker (M) size is 2000 bp; **B**: The double-enzyme digestion of pEGFP-N1 vector, Marker (M) size is 8000 bp; **C**: The PCR amplification of the overexpression vector Verification, Marker (M) size is 8000 bp; **D**: The verification of the overexpression vector by double enzyme digestion, Marker (M) size is 8000 bp; **E**: The verification of the overexpression vector by Xho I single enzyme digestion, Marker (M) size is 8000 bp)

The results in Fig. 3 show that the pEGFP-N1-Msx2 overexpression vector has been successfully constructed.

### Consequences of *Msx2* gene overexpression and silencing in goose embryo dermal fibroblasts

**Fig. 4:**
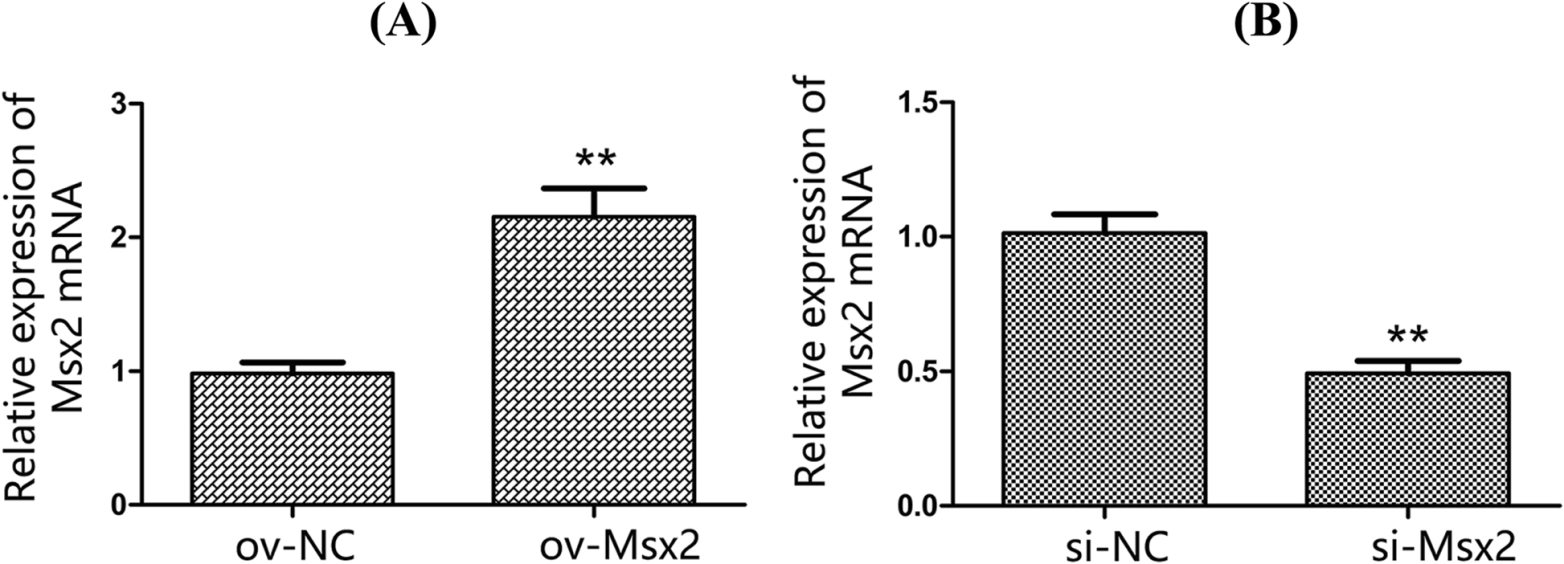
Overexpression and silencing of *Msx2* gene. (**A**: The pEGFP-N1-Msx2 transfection result; **B**: The si-Msx2 transfection result; Duncan multiple range test was used to determine statistical significance; **indicate Extremely significant difference, *P* < 0.01, *n* = 3)

The pEGFP-N1-Msx2 overexpression vector and si-Msx2 siRNA vector and transfecting goose dermal fibroblasts were constructed (Fig. 4A). At the mRNA level, the ov-Msx2 group significantly increased the expression level of *Msx2* gene at the mRNA level (*p* < 0.01). The expression level of *Msx2* gene in the si-Msx2 group was significantly lower than that in the si-NC control group (*p* < 0.01) (Fig. 4B), which indicates that si-Msx2 successfully interfered with the expression of *Msx2* gene. The above results show that pEGFP-N1-Msx2 and si-Msx2 have been successfully transfected into goose dermal fibroblasts and can be stably expressed, which lays the foundation for subsequent related research by promoting or inhibiting the expression of *Msx2* gene.

### Effects of *Msx2* gene on the viability of goose embryo dermal fibroblasts

**Fig. 5:**
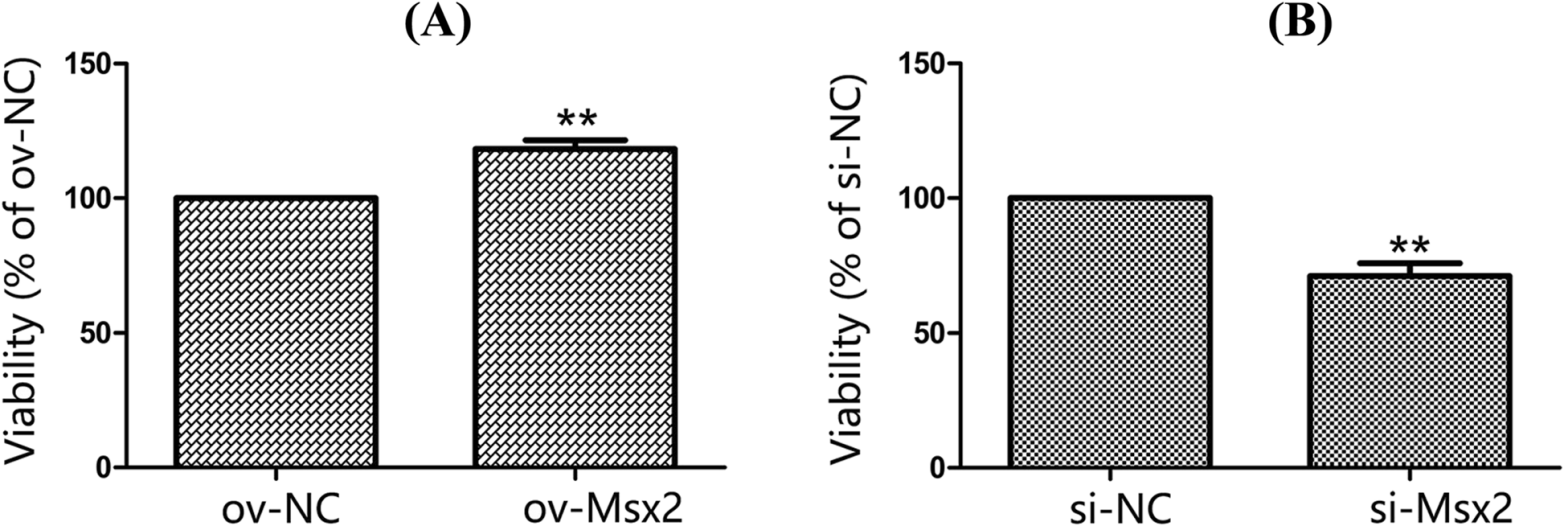
Proliferation of goose embryo dermal fibroblast. (**A**: The effect of pEGFP-N1-Msx2 on the viability of goose embryo dermal fibroblasts; **B**: The effect of si-Msx2 on the viability of goose embryo dermal fibroblasts; Duncan multiple range test was used to determine statistical significance; **indicate a very significant difference, *P* < 0.01, *n* = 3)

To explore whether the *Msx2* gene can affect the proliferation of goose embryo dermal fibroblasts, the MTT method was used to transfect pEGFP-N1-Msx2 and si-Msx2, respectively, the viability of goose embryo dermal fibroblasts was detected after changing the expression level of *Msx2* gene in cells. After pEGFP-N1-Msx2 treatment of goose embryo dermal fibroblasts, the cell viability was significantly increased (*p* < 0.01) (Fig. 5A); Meanwhile, compared with the si-NC group (Fig. 5B), the cell viability of the si-Msx2 group was significantly decreased (*p* < 0.01). It is speculated that *Msx2* gene may promote the proliferation of goose embryo dermal fibroblasts.

### Effects of *Msx2* gene on the proliferation and feather follicle development of goose embryo dermal fibroblasts

**Fig. 6:**
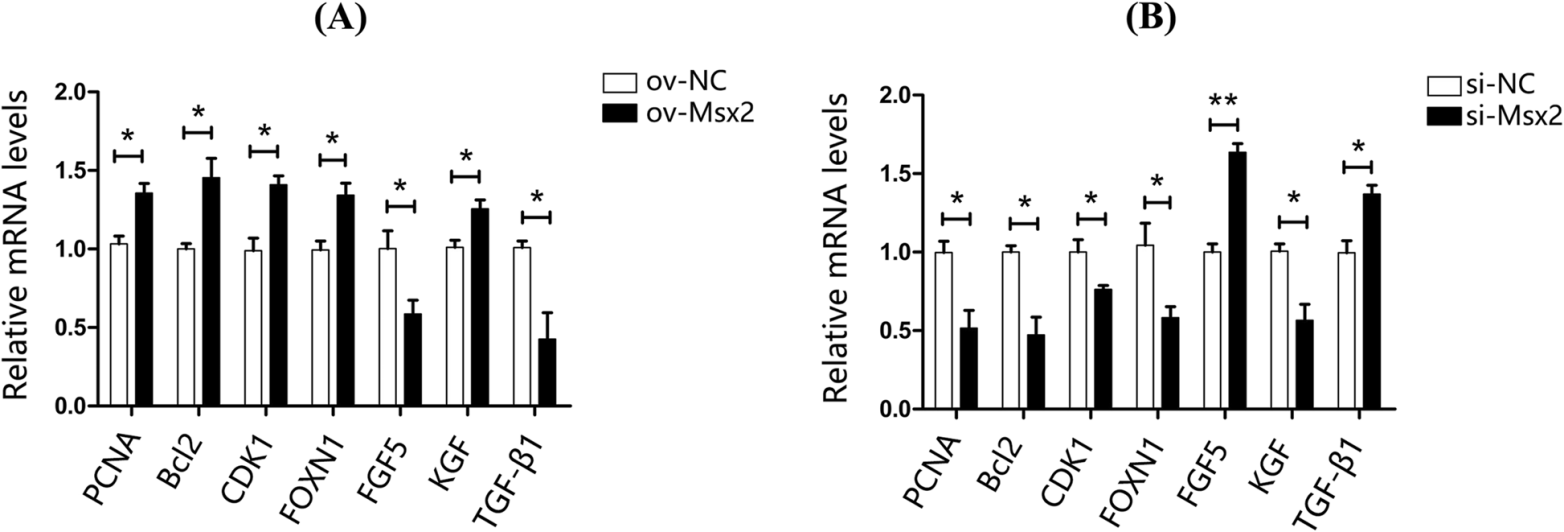
Relative expression of genes related to proliferation and feather follicle development in goose embryo dermal fibroblasts. (**A**: The effect of pEGFP-N1-Msx2 on the mRNA expression of goose dermal fibroblast-related genes; **B**: The effect of si-Msx2 on the mRNA expression of goose dermal fibroblast-related genes; Duncan multiple range test was used to determine statistical significance; *indicate significant difference, *P* < 0.05, *n* = 3)

In order to further clarify the effect of *Msx2* gene on the proliferation of goose dermal fibroblasts and the development of skin feather follicles, the Quantitative Real-time PCR method was used to detect the proliferation and apoptosis-related genes (*PCNA*, *Bcl2*) and cell cycle-related genes (*CDK1*) of goose dermal fibroblasts, growth factors (*KGF*, *TGF-β1*) secreted by dermal fibroblasts, and mRNA expression levels of skin feather follicle growth and development-related genes (*FGF5*, *FOXN1*). The test results showed that after pEGFP-N1-Msx2 was transfected into goose dermal fibroblasts, compared with the control group ov-NC, the gene expressions of *FGF5* and *TGF-β1* were significantly down-regulated (*P* < 0.05), *PCNA*, *Bcl2*, *CDK1*, *FOXN1* and *KGF* gene expression were significantly up-regulated (*P* < 0.05) (Fig. 6A); si-Msx2 significantly increased the expression of *TGF-β1* in goose dermal fibroblasts (*P* < 0.05), significantly increased the expression of *FGF5* (*P* < 0.01), and significantly decreased the expression of *PCNA*, *Bcl2*, *CDK1*, *FOXN1* and *KGF* genes (*P* < 0.05) (Fig. 6B).

### Detection and typing of SNPs of *Msx2* gene in Hungarian white goose

The Hungarian white goose *Msx2* gene exon SNP was screened by high-throughput sequencing, and 11 single nucleotide mutation sites were detected (Table 1). T71G, G78C, G121A and G140T are missense mutation. G84T, G1679T, G1853A are synonymous mutations. The genotype frequency and allele frequency were calculated (Table 2).

**Table 1:** *Msx2* gene exon SNPs information

SNP loci	Mutation site	Amino acid
T71G	missense mutation	L-R (Leucine-Arginine)
G78C	missense mutation	K-N (Lysine-Asparagine)
G84T	synonymous mutation	I (Isoleucine)
G121A	missense mutation	E-K (Glutamate-Lysine)
G140T	missense mutation	R-L (Arginine-Leucine)
C1679T	synonymous mutation	R (Arginine)
G1853A	synonymous mutation	P (Proline)
C2127T	nonsense mutation	–
A2229del_A	nonsense mutation	–
A2247ins_A	nonsense mutation	–
G2324ins_G	nonsense mutation	–

**Table 2:** Genotype frequency and allele frequency of missense SNP loci in *Msx2* gene

SNP loci	Genotype frequency (%)			Allele frequency (%)	
T71G	TT	TG	GG	T	G
	81.93%	16.87%	1.2%	90.37%	9.63%
G78C	GG	GC	CC	G	C
	0%	2.41%	97.59%	1.21%	98.79%
G121A	GG	GA	AA	G	A
	0%	2.41%	97.59%	1.21%	98.79%
G140T	GG	GT	TT	G	T
	85.54%	14.46%	0%	92.77%	7.23%

### Genetic polymorphism analysis of missense SNP loci

The polymorphism analysis of each missense SNP loci is shown in Table 3. The homozygosity of each SNPs loci in the test population was high. The *PIC* were 0.1589, 0.0236, 0.0236 and 0.1251, all of which were low polymorphisms (*PIC* < 0.25).

**Table 3:** Genetic polymorphism analysis of each mutation

SNP loci	Ho	Ne	*PIC*
T71G	0.8293	1.2137	0.1589
G78C	0.9878	1.0123	0.0236
G121A	0.9878	1.0123	0.0236
G140T	0.8659	1.1431	0.1251

### Prediction of the G78C mutant protein structure

Taking the Msx2 protein sequence of Zhe-Dong White Goose published by NCBI as a reference, the primary and secondary structures of the protein were predicted. The isoelectric point of Msx2 protein is 10.40, the instability coefficient is 50.98, the fat coefficient is 74.83, and the overall average hydrophilicity is − 0.442, α-helix was 36.32%, β-turn was 3.98%, extended chain was 12.44%, and random helix was 47.26%. The primary and secondary structures of the G78C mutant are shown in Table 4.

**Table 4:** Changes in protein primary and secondary structures before and after mutation

Structure name	Before	After
Isoelectric point	10.40	10.37
Instability factor	50.98	51.36
Fat coefficient	74.83	74.83
Overall average hydrophilicity	−0.442	−0.440
α helix	36.32%	31.34%
*β* turn	3.98%	4.98%
Extended strand	12.44%	8.46%
Random coil	47.26%	55.22%

### Association analysis between feather follicle diameter and G78C loci in Hungarian white goose

**Fig. 7:**
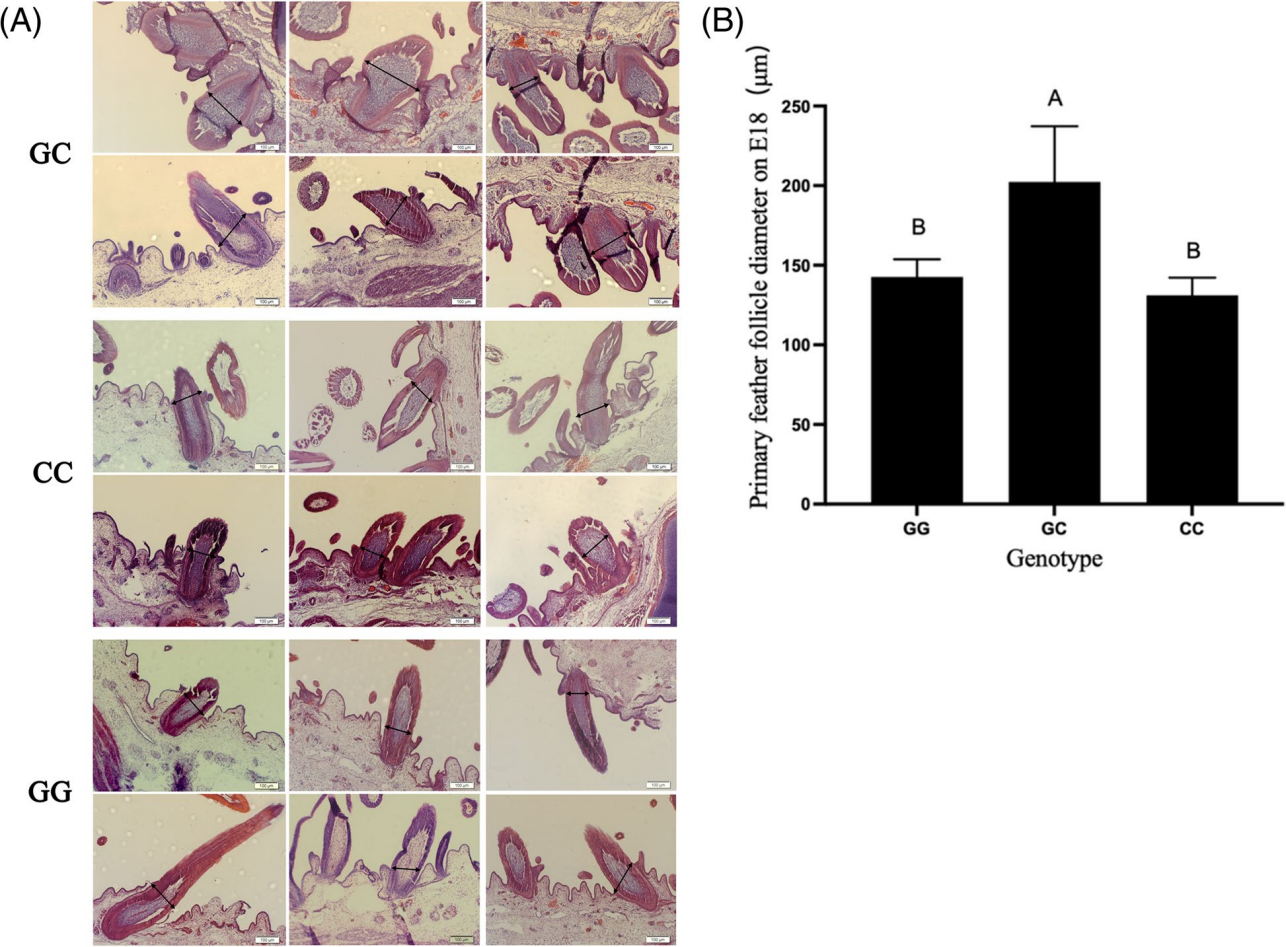
Measurement of primary feather follicle diameter of each genotype at G78C loci. (**A**: Tissue sections corresponding to different genotypes, Bar = 100 μm; **B**: Measurement of primary hair follicle diameter in E18 individuals with different genotypes. Duncan multiple range test was used to determine statistical significance; different letters indicate significant differences, *P* < 0.05, *n* = 6)

Genomic DNA was extracted from pre-collected embryonic blood, and primers specific for exon 1 of *Msx2* gene were designed for conventional PCR. And the PCR products were subjected to SNP detection by Sanger sequencing. The correlation analysis between the G78C loci and the primary feather follicle diameter on E18 was carried out (Fig. 7). It was found that the feather follicle diameter of the GC genotype at the G78C mutation was significantly longer than that of other genotypes.

## Discussion

The embryonic stage is the initial stage of the development of goose feather follicles, in which the density and number of feather follicles are fixed [11]. Therefore, research on the development of goose embryo feather follicles is of pioneering significance for the breeding of down varieties. The current research on the development of feather follicles in the embryonic stage of goose shows that E13–14 are the primitive stages of primary feather follicles, and E18 is the primitive stage of primary feather follicles, they are interconnected at E23–24; primary and secondary feather follicles are regularly arranged at E28 [12–14]. HE staining results of paraffin sections in this study showed that, at E13 epidermal structures and feather buds have formed, confirming this is the stage of primary folliculogenesis. At E18, the skin and epidermis at the base of the branch were invaginated, the top of the branch was cylindrical and filled with pith, and began to grow, it was observed that the spinous processes developed independently, and the diameter of the secondary follicles was smaller than that of the primary follicles, confirming that this was the developmental stage of the secondary follicles. At E23, primary follicles are evenly distributed on the back skin. At E28, the degree of invagination of the epidermis was deepened, and oval dermal papilla could be seen at the end of the burr, which proved that this period was maybe the main development period of the secondary follicle embryo.

The development of feather follicles is affected by a complex regulatory network, including Bmp, Hox and Wnt [14–16]. In this experiment, qPCR was used to detect the expression of *Msx2* gene at four key time points of feather follicle development during embryonic development. The expression of Msx2 gene was highest at E28, when the feather follicle entered a new postnatal growth stage, indicating that Msx2 gene plays an important role in the growth stage of the feather follicle*.*

Histological analysis showed that overexpression of the *Msx2* gene in mice resulted in thickening of the basal epidermal and hyperkeratosis [7]. However, *Msx2*-deficient mice exhibited progressive feather loss, whereas *Msx2* deletion shortened the anagen phase but prolonged the catagen and telogen phases of the mouse feather follicle development cycle [15]. Noveen [16] found that the *Msx2* gene can further develop epithelial cells during the morphogenesis of chicken embryo skin appendages and promote the development of epithelial cells into skin appendages. Similarly, in this study, *Msx2* gene was selected as the target gene to preliminarily explore its related regulatory mechanism in the development of goose feather follicles. Proliferating cell nuclear antigen (*PCNA*) acts as an accessory that affects DNA synthesis in cells. The *PCNA* gene is abundantly expressed in proliferating cells and weakly expressed in quiescent cells [17]. The *Bcl2* gene is located in the mitochondrial outer membrane of living cells and has an inhibitory effect on cell apoptosis, and its downregulation can induce apoptosis [18]. Cyclin-dependent kinase 1 (*CDK1*) is an important central regulator that enables cells to enter mitosis from G2 phase and plays a regulatory role in the cell cycle [19]. Therefore, the changes in *PCNA*, *Bcl2* and *CDK1* gene expression can be used to illustrate the effect of *Msx2* gene overexpression or silencing on the proliferating cells. In this study, pEGFP-N1-Msx2 led to increased *Msx2* gene expression (*P* < 0.01), increased cell viability (*P* < 0.01), and significant increased *PCNA*, *Bcl2* and *CDK1* genes expression (*P* < 0.05); The above results suggest that *Msx2* gene can act on goose dermal fibroblasts, inhibit the apoptosis of dermal fibroblasts, and promote the proliferation of dermal fibroblasts.

Feather follicles formation depends on signaling relationship between dermal cells and epidermal cells. Dermal fibroblasts exist in animal skin tissue and can secrete a variety growth factor, such as transforming growth factor beta (*TGF-β*) and keratinocyte growth factor (*KGF*). *KGF* gene, also known as *FGF7*, belongs to the *FGFs* gene family and is an epithelial-specific growth factor that can specifically stimulate the proliferation of epithelial cells such as keratinocytes [20]. Li [21] found that the *KGF* gene exists in the inner root sheath of mouse feather follicles and may play a regulatory role. Since the stromal cells of the inner root sheath produce the feather shaft and *KGF* receptors are present on the stromal cells, it is speculated that *KGF* may promote feather growth. TGF-β includes three subtypes of *TGF-β1*, *TGF-β2* and *TGF-β3*. Among them, TGF-β1, as a functional cytokine, has an inhibitory effect on epithelial cells; TGF-β1 inhibit cell proliferation, promote cell apoptosis, and make feather follicles enter the growth phase. In the in vitro co-culture system established by Inui, androgens inhibited the growth and development of epithelial cells by inducing the expression of *TGF-β1* in human dermal papilla cells with androgenetic alopecia [22]. The results of this study showed that overexpression of *Msx2* gene in goose dermal fibroblasts significantly down-regulated (*P* < 0.05) the expression of *FGF5* and *TGF-β1*, and significantly up-regulated (*P* < 0.05) the expression of *FOXN1* and *KGF*. The expression of *TGF-β1* was significantly up-regulated (*P* < 0.05), the expression of *FGF5* was significantly up-regulated (*P* < 0.01), and the expression of *FOXN1* and *KGF* genes was significantly down-regulated (*P* < 0.05). It is suggested that *Msx2* gene may inhibitory the expression of *FGF5* and *TGF-β1*, and promote the expression of *FOXN1* and *KGF*. It is speculated that the *Msx2* gene may act on goose dermal fibroblasts and participate in regulating the growth and development of goose feather follicles. The specific regulatory process may be through regulating the expression levels of growth factors (such as *KGF*, *TGF-β1*) secreted by goose dermal fibroblasts, thereby affecting the proliferation and differentiation of epithelial cells, thereby regulating the appearance and development of goose dermal fibroblasts. This experiment lays the foundation for the regulation of goose feather follicle development by *Msx2* gene, but the specific mechanism of *Msx2* gene regulation of goose feather follicle growth and development still need follow-up research.

SNP is an important genetic marker with the characteristics of large number, strong dimorphism, good genetic stability, and easy automatic detection [23]. A total of 11 SNPs of *Msx2* gene were screened in the Hungarian white goose population. T71G, G78C, G21A, G140T belong to missense mutations. He, Ho and PIC are all important parameters for measuring population genetic polymorphisms [24]. The heterozygosity of the selected population observed in this experiment is close to the expected heterozygosity, which means that the species is less affected by factors such as external environmental selection and inbreeding, and the population is in a state of genetic equilibrium [25]. At the same time, the *PIC* of each locus is less than 0.25, and the polymorphism is low, which indirectly indicated that the protection measures for the introduced Hungarian white goose were better, and the population purity was higher.

At present, reports on the genetic polymorphism of *Msx2* gene in livestock and poultry are rare. Liu [26] conducted a genome-wide association analysis on the quality traits of hen eggs at 70 and 80 weeks of age and found that *Msx2* was significantly correlated with embryonic development and egg production. However, the relationship between this gene mutation and down-production Performance is not clear. Proteins are macromolecular substances necessary to maintain life. Different proteins have different structures and can produce different functions [27]. With the continuous progress of bioinformatics, bioinformatics tools have been widely used in human medicine and agriculture [28, 29]. In this experiment, the primary and secondary structures of proteins before and after G78C mutation were predicted, the results of primary and secondary structure prediction showed that after G78C mutation, the isoelectric point, the ratio of α helix and the ratio of extended chains all decreased; the instability coefficient, the total average hydrophilicity, the ratio of β turn and random coil all increased, these all suggest that protein stability is reduced. Correlation analysis between each genotype of the G78C mutation site and the diameter of primary feather follicles at E18 revealed that the diameter of primary feather follicles of individuals with GC genotype was significantly higher than that of other genotypes, which provided a potential molecular marker for the breeding of downy goose breeds.

## Conclusions

*Msx2* gene may be involved in inducing spiking initial signals. It can inhibit the apoptosis of goose dermal fibroblasts, promote the proliferation of dermal fibroblasts, and regulate the expression of growth factors secreted by dermal fibroblasts, thereby affecting the proliferation and differentiation of epithelial cells, thereby participating in the growth and development of dermal fibroblasts control. The primary feather follicle diameter of the GC genotype at the G78C mutation site was significantly larger than that of other genotypes, which may be a potential feature of breeding molecules.

## Methods

### Experimental animals

The Hungarian white goose population raised in the provenance base of Jilin Agricultural University was selected, and the eggs of this population were collected and hatched according to routine procedures.

### Sample collection

83 Hungarian white geese were cryopreserved by blood sampling from the saphenous vein. 80 Hungarian white goose eggs are used for hatching. Dorsal skin and tissue were collected on embryonic days 13, 18, 23 and 28, and embryonic blood was collected on embryonic day 13. Use the above samples for subsequent testing.

### Tissue embedding and sectioning

The extracted goose back tissue pieces were placed in 4% paraformaldehyde and rinsed with tap water for 12 h. This was followed by a gradient of alcohol and xylene. The tissue blocks treated above were treated twice with low-melting wax and high-melting wax, respectively. Finally, use a tissue embedding machine to complete the embedding. Serial sections were performed using a microtome, unrolled in 45 °C water, picked up with adhesive slides and dried in a 60 °C oven.

### HE staining

Sections were immersed in xylene for dewaxing twice, 5 min each time. Then soak in 100, 90, 70% ethanol and distilled water for 2 min each. Dyeing was carried out according to the instructions of the HE staining kit, and finally the slides were sealed with neutral resin glue and observed under a microscope after drying.

### Extraction of total RNA and cDNA synthesis

1. Cells: Add Tripure Reagent to the washed culture plate and collect the lysate into EP tubes; Tissue: After the tissue was minced, 0.3 mL of Tripure Reagent was added, homogenized on an ice box, and the lysate was collected.
2. Centrifuge at 12,000×g for 5 min at 4 °C, collect the supernatant, add 200 μL of 1-bromo-3-chloropropane, mix well, stand at room temperature for 10 min, centrifuge again, and take the upper aqueous phase.
3. Add an equal volume of isopropanol, stand at room temperature for 10 min, centrifuge at 12,000×g for 10 min at 4 °C, discard the supernatant, add 1 mL of 75% ethanol to wash the RNA precipitate, invert and mix, 4 °C, 7500×g Discard the supernatant after centrifugation for 5 min.
4. Instantaneous centrifugation, discard the supernatant, and let it dry in the ultra-clean table for 10 minutes. Add 60 μL of DEPC-treated water to dissolve the pellet. The RNA concentration and OD value were detected by ultra-micro spectrophotometer.
5. Take 800 ng RNA, add DEPC water and 10x Loading Buffer and mix well, and perform gel electrophoresis with 1% agarose.
6. cDNA was synthesized using MonScript™ RTIII Super Mix with dsDNase (Two-Step) (Monad Biotech Co., Ltd., China) and RNA template in a total volume of 40 μL.

### Quantitative real-time PCR

The primer sequences are shown in Table 5. The Quantitative Real-time PCR experiments were performed in an ABI 7500 PCR machine (ThermoFisher, USA) using ABP™ SYBR™ Green (ThermoFisher, USA). Each treatment group contained three biological replicates and each sample was tested for relative expression three times. GAPDH was selected as the internal reference gene, and the gene expression level was calculated by the 2^-ΔΔCT^ method.

**Table 5:** Quantitative Real-Time PCR primer information

Gene	Species	Primer Sequence	Length
Msx2	Anser cygnoides domesticus	TTCCGCCAGAAGCAGTATCT ATAGGGAAAGGGAGGCTGAA	197
PCNA	Anser cygnoides domesticus	CAGCCATATTGGTGATGCAG GGTCAGTTGGACTGGCTCAT	166
Bcl2	Anser cygnoides domesticus	CCTGGATGACCGAGTACCTG ATAAGCGCCAAGAGTGATGC	182
CDK1	Anser cygnoides domesticus	GAAGTCGTGACGCTGTGGTA TTGTTGGGTGTCCCTAAAGC	188
KGF	Anser cygnoides domesticus	AGTGGCAATCAAAGGAGTGG TGTGTCCATTTTGCAGAAGC	153
TGF-β1	Anser cygnoides domesticus	GACCTGCAGTGGAAGTGGAT GCCCCACGTAGTAGACGATG	202
FGF5	Anser cygnoides domesticus	ATCGGCTCCTCAGAAAGTGA TATGCGATACTCGGCAACAG	157
FOXN1	Anser cygnoides domesticus	GCTTCGGACACTCTGGAAAG CTGGGGCTGTGAGAAGACTC	164
β-actin	Anser cygnoides domesticus	GCATGCCACACCGTGCCCATCTATGAG AAGCTTGGCCATCTCCTGCTCGAAGT	205

### Cell isolation and cultural

Skin was excised from the back of Hungarian white goose on embryonic day 28. In a sterile environment, wash skin samples 3 times with D-Hank’s solution, transfer to a 35 mm cell culture dishes, add 3 mL of 2.5% disease II neutral protease in the dark, and t wrap the dishes with aluminum foil. Refrigerate overnight at 4 °C for complete digestion; the skin dermis of the skin is separated from the epidermis, Trypsinize at 37 °C for 1 h; filter after digestion in complete medium (using DMEM/F12 + 10% fetal bovine serum), centrifuge filtrate at 1100 rpm for 6 minutes, discard resuspended supernatant, repeat filtration and centrifugation for 4 times; resuspended in complete medium, adjusted to viable cell density, seeded into cell culture plates, and placed in a 5% CO2 (37 °C, saturated humidity) incubator for 24 hours. The complete medium was replaced to remove non-adherent cells.

### Giemsa staining and immunofluorescence of cells

Giemsa staining of cells: methanol-fixed dermal fibroblasts were covered with Giemsa working solution, stained at room temperature for 25 minutes, rinsed slowly with distilled water, and observed under a microscope after drying.

Cell immunofluorescence: When the growth density of goose dermal fibroblasts cultured in the 24-well cell culture plate reached 50%, the medium was removed, and the cells were washed 3 times with PBS for 4 min each; cells were fixed with pre-cooled 10% formalin at room temperature 20 min. Rinse after 10 min; permeabilize cells with 0.2% Triton X-100 for 10 min, rinse; block with 1% goat serum for 1 h, remove the blocking solution, add primary antibody (rabbit anti-vimentin antibody, 1:100 dilution), 4 Incubate at °C for 15 h, rinse clean.

### Construction of Msx2 gene overexpression vector and plasmid transfection

Primers (Msx2-F:5′-ATGGCTTCTCCTTCCAAGGCG-3′ and Msx2-R:5′-CTGATCTTCTTAGGATAAGTGGTACATGC-3′) were designed according to the CDS sequence of the *Msx2* gene obtained by transcriptome sequencing, and PCR was carried out using the goose E28 skin. cDNA was used as the template for amplification. After electrophoresis detection, the target fragment was recovered with a gel recovery kit, and PCR was performed using *Msx2* gene homologous recombination primers (Msx2-S:5′-CTACCGGACTCAGATCTCGAGATGGCTTCTCCTTCCAAGGC-3′ and Msx2-AS: 5′-ATGGTGGCGACCGGTGGATCCCGGGATAAGTGGTACATGCTATATC-3′). According to the instructions of the non-ligase-dependent rapid single-fragment cloning kit (Nanjing Vazyme), the purified and recovered *Msx2* gene fragment was ligated into the pEGFP-N1 linear vector, and the recombinant product was digested with BamH I and Xho I enzymes. Transformed into DH5α competent cells. According to the instructions of the endotoxin-free plasmid medium extraction kit (Beijing Biolab), the recombinant plasmid was extract by shaking the anti-pseudonym, and the overexpression vector was successfully constructed by sequencing and PCR. No verification. Named pEGFP-N1-Msx2. When the density of goose dermal fibroblasts in the 24-well cell culture plate was 2*10^5^ cells/well, and the cell confluence rate was 70%, the empty pEGFP-N1 plasmid was recombined with pEGFP-N1-Msx2 transfection reagent according to the instructions. Plasmid for cell transfection.

### siRNA transfection

*Msx2* gene specific siRNA and si-NC were designed and synthesized by Jiying Biotechnology Co., Ltd. (Shanghai). Si-Msx2 sequence: 5′-CCTCGGTTAAATCGGAGAA-3′, negative control (si-NC) sequence: 5′-TTCTCCGAACGTGTCACGT-3′ According to the siRNA product instruction manual and transfection reagent instructions, 50 nM si-Msx2 and si-NC were respectively Transient transfections were performed.

### Cell viability assay

Dermal fibroblasts in logarithmic growth phase and successfully transfected with si-NC, si-Msx2, ov-NC, and ov-Msx2 were seeded in a 96-well cell culture plate at a density of 4 × 104 cells/ml. After wall growth, wash with PBS, add 90 μL of fresh medium and 10 μL of 0.5% MTT, incubate for 3 h, discard the medium, add 100μLDMSO, shake at low speed in the dark for 8 min, and measure the absorbance value (OD490). Six parallel controls (*n* = 6) were set up for each sample. Cell viability (%) = [A (vehicle) - A (blank)] / [A (control group) - A (blank)] X 100. A (Vector): Refers to the absorbance of cells successfully transfected with pEGFP-N1-Msx2 or si-Msx2, MTT reagent, and culture medium in the wells; A (Control group): Refers to wells containing successfully transfected pEGFP-N1 or si-Msx2 cells Absorbance of si-NC in cells, MTT reagent, and medium; A (blank): refers to the absorbance when the well contains only MTT reagent and medium without any cells.

### Extraction of genomic DNA

Genomic DNA was extracted using the Complete Gold Easy Pure Blood Genomic DNA Kit. Place 20 μL blood sample in a centrifuge tube, add RNase and Proteinase K, aspirate, and mix, then add 500 μL BB3 solution, vortex for 15 s, incubate at room temperature for 10 min, then centrifuge at 12,000 g for 1 min, add 500 μL CB3 Add WB3 to both batches, centrifuge at 1200 g for 30 sec, discard the centrifuge, transfer the spin column to a new centrifuge tube, Add 50 μL of pre-warmed EB solution, centrifuge at 1200 g for 1 minute to elute the DNA, collect in a 200 μL centrifuge tube, titrate the DNA concentration and OD value, and store at − 20 °C in a spare refrigerator.

### Multiplex PCR and sequencing

A set of 45 SNP target sites was designed. The library was made by a two-step PCR process. The first round of PCR reaction was as follows: DNA (10 ng/μL) 2 μL; Amplicon PCR forward primer mix (10 μM) 1 μL; Amplicon PCR reverse primer mix (10 μM) 1 μL; 2 × PCR Ready Mix 15 μL (total 25 μL) (Kapa HiFi Ready Mix). The plate was sealed and PCR was performed in a thermal instrument analyzer (BIO-RAD, T100TM) using the following procedure (Table 6):

**Table 6:** first round PCR reaction procedure

Temperature	Duration	Cycle
98 °C	5 min	
98 °C	30s	8×
50 °C	30s	
72 °C	30s	
98 °C	30s	25×
66 °C	30s	
72 °C	30s	
72 °C	5 min	
4 °C	Hold	

The PCR products were checked using electrophoresis in 1% agarose gels in TBE buffer (Tris, boric acid, EDTA) stained with ethidium bromide (EB) and visualized under UV light. Then we used AMPure XP beads to purify the amplicon product. After that, the second round PCR was performed. PCR reaction was set up as follows: DNA (10 ng/μL) 2 μL; universal P7 primer with barcode (10 μM) 1 μL; universal P5 primer (10 μM) 1 μL; 2× PCR Ready Mix 15 μL (total 30 μL) (Kapa HiFi Ready Mix). The plate was sealed and PCR performed in a thermal instrument (BIO-RAD, T100TM) using the following program (Table 7):

**Table 7:** second round PCR reaction procedure

Temperature	Duration	Cycle
98 °C	3 min	
94 °C	30s	5×
55 °C	20s	
72 °C	30s	
72 °C	5 min	

Then we used AMPure XP beads to purify the amplicon product. The libraries were then quantified and pooled. Paired-end sequencing of the library was performed on the HiSeq XTen sequencers (Illumina, San Diego, CA).

### Data QC and SNP calling

Raw reads are screened in two steps: 1) if the reads contain cut adapt (v 1.2.1), Removing the linker sequence. 2) use PRINSEQ-lite (v 0.20.3) to Remove reads 3′ to 5′ from low quality bases (Q < 20); the remaining clean data was mapped to the reference genome by BWA (version 0.7.13-r1126) using default parameters. Write a Perl script to calculate each genotype at the target site. Detection of genetic variation using Annovar (2018-04-16).

### Genetic polymorphism analysis

The genotype frequency, allele frequency, effective allele number (Ne), population genetic homozygosity (Ho) and polymorphism information content (PIC) of each SNP locus were calculated by Excel software.

Use the online software expasy (https://www.expasy.org/resources/protparam) to analyze the protein primary structure; use the online software SOPMA (https://npsa-prabi.ibcp.fr/cgi-bin/npsa_automat. pl? page = npsa_sopma) to predict and analyze the protein secondary structure of each haplotype before and after mutation in the coding region of *Msx2* gene.

## Supplementary Information

**Additional file 1.** The agarose gel electrophoresis diagram of the construction of the overexpression vector is shown in the fig. S1.

## References

[1] Yu M, Wu P, Widelitz RB, Chuong C-M (2002). The morphogenesis of feathers. Nature.

[2] Chen X, Bai H, Li L, Zhang W, Jiang R, Geng Z (2012). Follicle characteristics and follicle developmental related Wnt6 polymorphism in Chinese indigenous Wanxi-white goose. Mol Biol Rep.

[3] Kim B-K, Yoon SK (2013). Hairless down-regulates expression of Msx2 and its related target genes in hair follicles. J Dermatol Sci.

[4] Chen Y-H, Ishii M, Sun J, Sucov HM, Maxson RE (2007). Msx1 and Msx2 regulate survival of secondary heart field precursors and post-migratory proliferation of cardiac neural crest in the outflow tract. Dev Biol.

[5] Liu H, Jiang J, Gong Q, Tian H, Wang S, Zhang J, Pan Z, Liu X (2018). Knockdown of MSX2 gene inhibits the expression of enamel matrix proteins and the enamel mineralization in mouse ameloblasts. Chi J of Cell and Mole Immun.

[6] Taghiyar L, Hesaraki M, Sayahpour FA, Satarian L, Hosseini S, Aghdami N, Baghaban Eslaminejad M (2017). Msh homeobox 1 ()- and -overexpressing bone marrow-derived mesenchymal stem cells resemble blastema cells and enhance regeneration in mice. J Biol Chem.

[7] Jiang TX, Liu YH, Widelitz RB, Kundu RK, Maxson RE, Chuong CM (1999). Epidermal dysplasia and abnormal hair follicles in transgenic mice overexpressing homeobox gene MSX-2. The J of Invest Dermatol.

[8] Hughes MW, Jiang T-X, Plikus MV, Guerrero-Juarez CF, Lin C-H, Schafer C, Maxson R, Widelitz RB, Chuong C-M (2018). Msx2 supports epidermal competency during wound-induced hair follicle Neogenesis. The J of Invest Dermatol.

[9] Matsuda K (2017). PCR-based detection methods for single-nucleotide polymorphism or mutation: real-time PCR and its substantial contribution toward technological refinement. Adv Clin Chem.

[10] Rubin BE, Ree RH, Moreau CS (2012). Inferring phylogenies from RAD sequence data. PLoS One.

[11] Zhou P, Byrne C, Jacobs J, Fuchs E (1995). Lymphoid enhancer factor 1 directs hair follicle patterning and epithelial cell fate. Genes Dev.

[12] Liu C, Sello CT, Sui Y, Hu J, Chen S, Msuthwana P, Zhou Y, Wachiebine SK, Sun Y, Liu J (2020). Characterization of Embryonic Skin Transcriptome in at Three Feather Follicles Developmental Stages. G3.

[13] Liu C, Sello CT, Sun Y, Zhou Y, Lu H, Sui Y (2018). Transcriptome sequencing analysis of goose () embryonic skin and the identification of genes related to feather follicle morphogenesis at three stages of development. Int J Mol Sci..

[14] Sello CT, Liu C, Sun Y, Msuthwana P, Hu J, Sui Y (2019). De novo assembly and comparative transcriptome profiling of and geese Species' embryonic skin feather follicles. Genes (Basel)..

[15] Ma L, Liu J, Wu T, Plikus M, Jiang T-X, Bi Q, Liu Y-H, Müller-Röver S, Peters H, Sundberg JP (2003). 'Cyclic alopecia' in Msx2 mutants: defects in hair cycling and hair shaft differentiation. Development.

[16] Noveen A, Jiang TX, Ting-Berreth SA, Chuong CM (1995). Homeobox genes Msx-1 and Msx-2 are associated with induction and growth of skin appendages. The J of Invest Dermatol.

[17] Li W, Li C, Chen S, Sun L, Li H, Chen L, Zhou X (2018). Effect of inhibin a on proliferation of porcine granulosa cells in vitro. Theriogenology.

[18] Edison N, Curtz Y, Paland N, Mamriev D, Chorubczyk N, Haviv-Reingewertz T, Kfir N, Morgenstern D, Kupervaser M, Kagan J (2017). Degradation of Bcl-2 by XIAP and ARTS promotes apoptosis. Cell Rep.

[19] Diril MK, Ratnacaram CK, Padmakumar VC, Du T, Wasser M, Coppola V, Tessarollo L, Kaldis P (2012). Cyclin-dependent kinase 1 (Cdk1) is essential for cell division and suppression of DNA re-replication but not for liver regeneration. Proc Natl Acad Sci U S A.

[20] Yang K, Yin J, Sheng B, Wang Q, Han B, Pu A, Yu M, Sun L, Xiao W, Yang H (2017). AhR-E2F1-KGFR signaling is involved in KGF-induced intestinal epithelial cell proliferation. Mol Med Rep.

[21] Li X, Wang X, Bai L, Zhao P, Zhang M (2019). Exposure to 50 Hz electromagnetic fields enhances hair follicle regrowth in C57BL/6 mice. Exp Biol Med (Maywood).

[22] Inui S, Fukuzato Y, Nakajima T, Yoshikawa K, Itami S (2002). Androgen-inducible TGF-beta1 from balding dermal papilla cells inhibits epithelial cell growth: a clue to understand paradoxical effects of androgen on human hair growth. FASEB J.

[23] Vignal A, Milan D, SanCristobal M, Eggen A (2002). A review on SNP and other types of molecular markers and their use in animal genetics. Gen, Select, Evol: GSE.

[24] Qin Y, Shi G, Sun Y (2013). Evaluation of genetic diversity in Pampus argenteus using SSR markers. Genet Mol Res.

[25] Takezaki N, Nei M (1996). Genetic distances and reconstruction of phylogenetic trees from microsatellite DNA. Genetics.

[26] Liu Z, Sun C, Yan Y, Li G, Shi F, Wu G, Liu A, Yang N (2018). Genetic variations for egg quality of chickens at late laying period revealed by genome-wide association study. Sci Rep.

[27] Anfinsen CB (1973). Principles that govern the folding of protein chains. Science.

[28] Jumper J, Evans R, Pritzel A, Green T, Figurnov M, Ronneberger O, Tunyasuvunakool K, Bates R, Žídek A, Potapenko A (2021). Highly accurate protein structure prediction with AlphaFold. Nature.

[29] AlQuraishi M (2021). Machine learning in protein structure prediction. Curr Opin Chem Biol.

