# Supplementary figures and images for: Regulation of feather follicle development and Msx2 gene SNP degradation in Hungarian white goose
Source: BMC Genomics. 2022 Dec 12;23:821. doi: 10.1186/s12864-022-09060-z (PMC9743523; doi:10.1186/s12864-022-09060-z)

## Slide 1
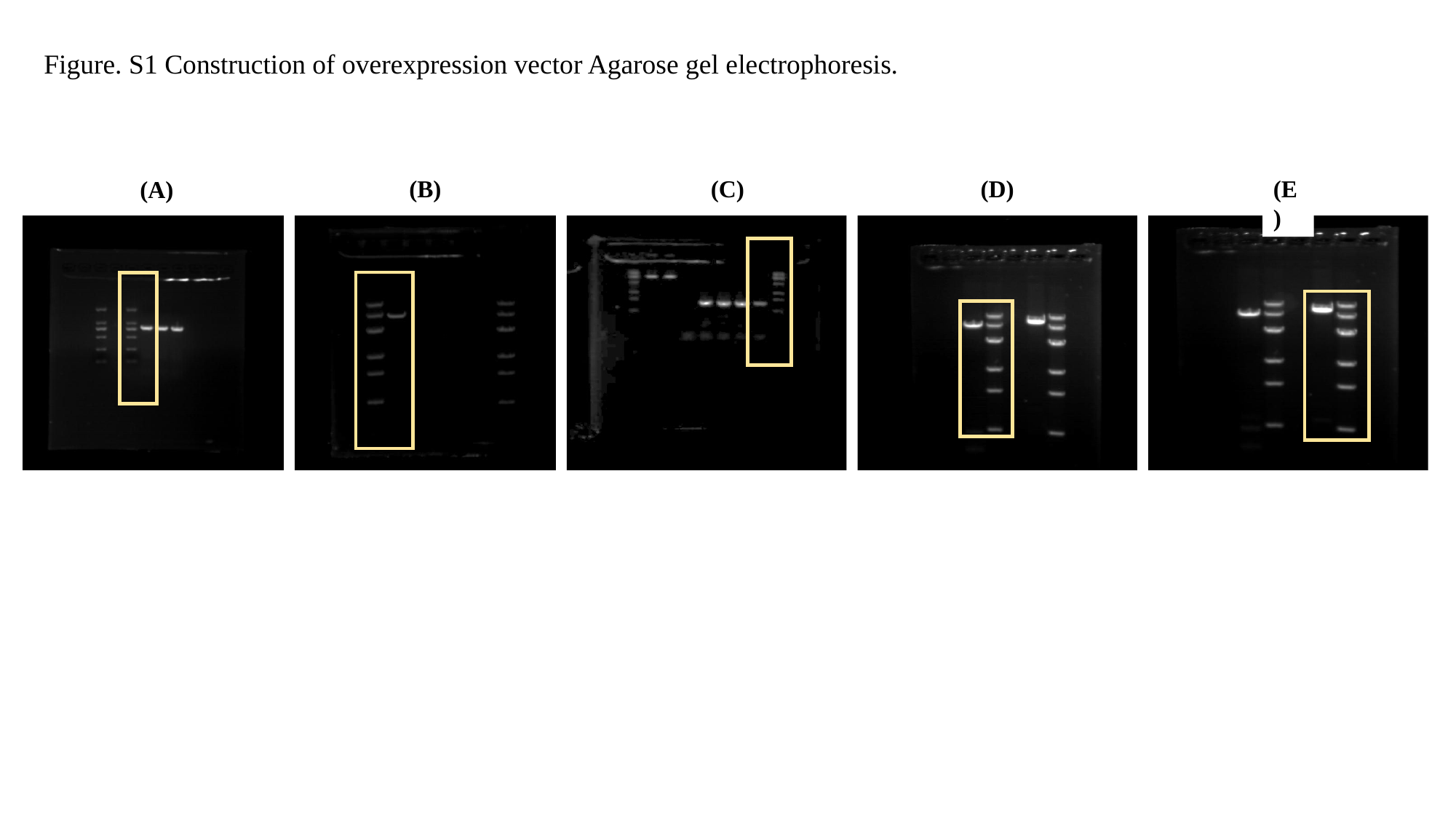

Figure. S1 Construction of overexpression vector Agarose gel electrophoresis.
(B)
(D)
(E)
(C)
(A)

Supplement: Supplementary file 1 — Additional file 1. The agarose gel electrophoresis diagram of the construction of the overexpression vector is shown in the fig. S1. [file 12864_2022_9060_MOESM1_ESM.pptx]
